# Silk Nanofibril-Palygorskite Composite Membranes for Efficient Removal of Anionic Dyes

**DOI:** 10.3390/nano13020247

**Published:** 2023-01-06

**Authors:** Xu-rui Wang, Zhe-yi Meng, Xue-fen Wang, Wei-long Cai, Ke Liu, Dong Wang

**Affiliations:** 1 State Key Laboratory for Modification of Chemical Fibers and Polymer Materials, College of Materials Science and Engineering, Donghua University, Shanghai 201620, China; 2 Qingyuan Innovation Laboratory, Quanzhou 362801, China; 3 Key Laboratory of Textile Fiber and Products, Ministry of Education, Hubei International Scientific and Technological Cooperation Base of Intelligent Textile Materials and Application, Wuhan Textile University, Wuhan 430200, China

**Keywords:** silk nanofibrils, palygorskite, genipin, water purification, anionic dyes

## Abstract

To develop membrane materials with good performance for water purification that are green and low cost, this work reports an organic–inorganic composite membrane composed of silk nanofibrils (SNFs) and palygorskite (PGS). To improve the stability of the the composite membrane, genipin was used as a crosslinking agent to induce the conformational transition of SNF chains from random coils to β-sheets, reducing the swelling and hydrolysis of the membrane. The separation performance can be adjusted by tailoring the component ratio of the nanomaterial. The results showed that these membranes can effectively remove anionic dyes from water, and they exhibit excellent water permeability. The SNF-based membrane had strong mechanical and separation properties, and the PGS could tune the structure of composite membranes to enhance their permeability, so this green composite membrane has good prospects in water treatment and purification applications.

## 1. Introduction

Owing to the rapid development of the global industry, a large number of heavy-metal ions, organic dyes, and other pollutants are discharged into water sources [1]. Containing a large number of organic pollutants that are toxic and hard to degrade [2,3], dye wastewater poses a great threat to the health of humans and animals and can lead to physiological malformations, cancer, and other serious diseases [4,5]. In the production and application of dyes, 10~30% are discharged without any treatments [6], which endangers the natural aqueous environment and the ecological balance. As such, techniques and related materials for the removal of dyes in wastewater are urgently desired.

At present, common methods of water treatment, such as adsorption [5,7], catalytic oxidation [8,9], flocculation [10,11], and membrane separation [12,13], have been extensively applied. Among these, membrane technology, as a pure physical separation process, needs to neither decompose components that produce new substances nor add chemicals, and it has the advantages of simple operation, high separation efficiency, easy scaling-up, and low energy consumption [14]. As a promising technology for water treatment and purification, membrane technology still faces a challenge regarding the trade-off between selectivity and permeability. Ultrafiltration membranes generally have a high flux, but due to the relatively large membrane pore size, the rejection of substances with a diameter of less than 10 nm has little effect [15]. On the other hand, despite the high retention of nanofiltration to small molecules, high operating pressure, membrane pollution, and other problems have always limited its application in the direction of water purification [16], so it has been difficult to achieve the ideal goals for membrane technology in the field of water purification. As such, the membranes’ permeability still has great potential for achieving further progress. Therefore, it is urgent to find a new way to overcome the above deficiencies.

Natural materials with fine nanostructures bring inspiration for novel membrane designs to break through the limitation of the trade-off between selectivity and permeability. Because of their unique properties, natural materials have a wide range of applications in membrane technology. Functionalized cellulose nanofibers (CNFs) embedded in a polymer matrix create hydrophilic nanochannels that significantly increased membrane flux by seven times and effectively rejected bovine serum albumin [17]. The functional bacterial cellulose membranes can realize the separation of oil and water at 0.1 MPa [18]. Silk is one of the earliest known natural polymer materials with the largest yield, and its main proteinic component, fibroin, has also been intensively investigated [19]. Liquid-stripped silk nanofibrils (SNFs), composed of fibroins, can be used to produce high water flux filtration membranes with high separation performance for dyes, proteins, and nanoparticles with certain mechanical strength [14,20]. However, similar to other filtration membranes with a biomaterial as the main raw material, the original SNF membrane pore size was not adjustable [21], which required more operating pressure and was not conducive to practical application. With an increase in the selective layer thickness deposited on substrates, the path of a solvent through the membrane became longer, and the porous structure inside the membranes would also hinder the flow of solvent, so the permeation rate of the solvent decreased significantly. These problems can be partly solved by introducing some kind of inorganic material into the membrane to optimize the pore structure of membranes. For example, hydroxyapatite (HAP) [22] and molybdenum disulfide (MoS_2_) nanosheets [23] have been applied to silk protein composite membranes, but these synthetic nanomaterials are expensive, which is not convenient for large-scale use in the future [22,24]. Palygorskite has a layered crystal structure, a large specific surface area, excellent pore volume, and abundant free nanochannels, which give it excellent adsorption properties, and provide a material platform for the preparation of membrane materials with unique properties [25]. Thus, it is promising and feasible to utilize the two natural materials and combine their excellent properties to explore the cost-saving and environmentally friendly hybrid nanocomposite membrane of protein fiber and inorganic minerals.

To sum up, in some natural one-dimensional materials, silk fiber has an anti-parallel β-sheet structure, so it has excellent mechanical properties and can be widely used in the for environmental, energy, and other purposes, but these properties were shown to decrease after degumming [26]. Therefore, crosslinking is a necessary modification strategy for the development of promising and more stable natural material composite membranes. Genipin is a natural crosslinking agent with excellent properties, especially cytotoxicity. Compared with the commonly used crosslinking agent glutaraldehyde, genipin is significantly less toxic [27,28]. Genipin, as a crosslinker, induces the conformational transition of SNF chains from random coil to β-sheet, resulting in good mechanical properties and reduced expansion degree. These characteristics make genipin an ideal crosslinking agent for silk fibroin membrane preparation. Notably, genipin reacts slowly [29], which prevents excessive crosslinking and gel formation during membrane preparation. Therefore, among the many crosslinking agents, this indicates that genipin is a suitable and safe one.

**Figure 1:**
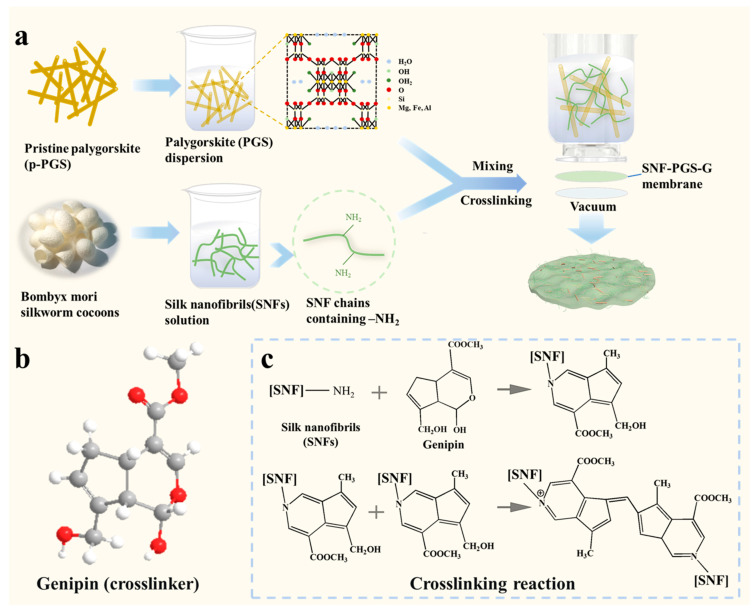
Preparation technology and crosslinking mechanism of mixed dimensional SNF-PGS-G composite membrane: (**a**) schematic diagram of composite membrane preparation; (**b**) structural formula of genipin; (**c**) crosslinking mechanism of SNF and genipin. Reprinted with permission from Ref. [30].

In this work, we report for the first time an economical and green composite membrane prepared from one-dimensional SNFs and inorganic mineral palygorskite with genipin crosslinking for water filtration. The SNF-PGS-G membrane was prepared by a simple vacuum filtration process, and the membrane preparation conditions were easy to control (as shown in Figure 1). By using genipin, which is much less bio-toxic than a traditional crosslinker, the β-sheet structure of the silk fibroin can be converted, which could improve the water resistance and stability of the membrane. The permeability and separation properties of SNF-PGS-G membranes were investigated for various dyes. The results showed that the membranes had excellent separation of different anionic dyes while maintaining a high level of permeability, compared with a traditional nanofiltration membrane.

## 2. Materials and Methods

### 2.1. Chemicals and Materials 

Bombyx mori silkworm cocoons were collected from Shanxi province in China. Palygorskite clay was purchased from Changzhou Dingbang Mineral Products Technology Co., Ltd. (Changzhou, China). NaHCO_3_ (≥99.5%) and LiBr (99.5%) were purchased from Aladdin Co., Ltd. (Shanghai, China). Nylon 6 membranes (cutoff: 450 nm; diameter: 47 mm) were obtained from Tianjin Jinteng Experimental Equipment Co., Ltd. (Tianjin, China). Genipin (≥98%), Congo red (CR, Mw = 696.99 g mol^−1^, BS), acid fuchsin (AF, Mw = 585.54 g mol^−1^, BS), direct red 80 (DR80, Mw = 1373.07 g mol^−1^, BS), brilliant blue G (BBG, Mw = 854.02 g mol^−1^, BS), methyl blue (MB, Mw = 799 g mol^−1^, BS), acid red94 (AR, Mw = 1017.64 g mol^−1^, BS), and Alcian blue 8GX (AB, Mw = 1298.86 g mol^−1^, BS) were purchased from Shanghai Titan Technology Co., Ltd. (Shanghai, China).

### 2.2. Preparation of SNF Solution

The silk nanofibril (SNF) solution was prepared as previously described [22]. First, Bombyx mori silkworm cocoons were chopped into suitable pieces to boil in three 30-min increments of 0.5% (*w/w*) NaHCO_3_ solution to remove sericin. Then, the degummed silk fibers were rinsed thoroughly with deionized water and allowed to air-dry at room temperature. Next, a 5% (*w/v*) solution of degummed silk fibroin (SF) was disolvedin an aqueous 9.3 M LiBr solution at 60 °C for 4 h. The obtained solution was then dialyzed with deionized water using a dialysis tube (Viskase, MWCO 3500 (Chicago, IL, USA)) at 4 °C for 72 h to yield SF solution with a protein concentration of ~2 wt %. The dialyzed solution was subsequently purified by centrifugation at 9800 rpm for 15 min. The concentration of the resulting solution was measured by weighing the residual solid after drying at room temperature to a constant weight. To produce silk nanofibrils (SNFs), this solution was diluted to 0.2 wt % SF in aqueous solution and incubated at 60 °C over 7 days. The solution was diluted to desired concentrations and stored in a refrigerator (2~4 °C) for further study.

### 2.3. Palygorskite Pretreatment and Functional Modification

The impurities of pristine palygorskite (p-PGS) samples were removed by washing in acid aqueous solutions and followed by drying and sintering. The p-PGS was calcined at 400 ℃ for 5 h, cooled to room temperature, weighed, and added into 4 mol L^−1^ hydrochloric acid solution, and then treated in ultrasonic bath for 30 min and stirred for 4 h. With deionized water to no chloride ions, 120 °C drying, and grinding sieve reserve use, hydrochloric acid treatment of palygorskite (PGS) was obtained. Palygorskite suspension was prepared by dispersing the acidified palygorskite (PGS) powder in distilled water.

### 2.4. Preparation of SNF/PGS Blended Precursor Solution

In this study, SNF solution (0.1% *w*/*v*) and PGS suspension (0.02% *w*/*v*) were mixed to prepare a blended precursor solution. SNF and PGS with different volume ratios (set as 4:1, 3:1, 3:2, and 1:1) were first mixed to obtain different SNF-PGS mixtures, which were labeled as 20:1, 15:1, 15:2, and 5:1 according to their weight ratios. Genipin, as the crosslinking agent (0.025% *w*/*v*), was added to the blended solutions under constant stirring at room temperature until complete dissolution. The crosslinking reaction was carried out for 3, 6, 12, 18, and 24 h at 37 °C. After the crosslinking reaction, mixed solutions of SNF-PGS-G with different mass ratios were obtained.

### 2.5. Preparation of SNF-PGS-G Composite Membranes

The SNF-PGS-G composite membranes were prepared by vacuum filtering certain amounts of SNF-PGS-G mixtures onto a polyamide (PA) microfiltration membrane with a pore diameter of 450 nm. To induce the transition of SNF from a random coil to a β-sheet structure, which is insoluble in water, membranes were immersed in 90% (*v*/*v*) methanol aqueous solution for 30 min and then washed in distilled water and air dried. The same steps were used as a control to prepare pure SNF membranes without PGS. 

### 2.6. Evaluation of Crosslinking Degrees

The crosslinking degree of SNF membrane samples reflects the effect of genipin, and it could be characterized via the measurement of the rest of the amino groups in the SNF membranes with the ninhydrin assay [31,32]. In the ninhydrin assay, the sample was lyophilized for 24 h and was then weighed (3 mg). Subsequently, the lyophilized sample was heated with a ninhydrin solution (0.35% *w*/*v*) at 100 °C for 10 min, and the optical absorbance of the solution was recorded with a microplate reader (BioTek, Epoch, Winooski, VT, USA) at a wavelength of 570 nm. After the sample was heated with ninhydrin, the number of free amino groups in the test sample was proportional to the optical absorbance of the solution. 

Glycine solutions of certain concentrations were used as standards, and SNF-PGS membranes that were prepared without genipin were used as a control. Each sample was repeated three times with the same amount of membrane and water as a control. The degree of crosslinking of the sample was determined by Equation (1):

(1) $$Degree\;of\;crosslinking\;\left(\%\right)=\frac{\left[{\left(NH_{2}\right)}_{nc}-{\left(NH_{2}\right)}_{c}\right]}{{\left(NH_{2}\right)}_{nc}}\times 100$$

where *(NH*_2_*)_nc_* and *(NH*_2_*)_c_* are the mole fraction of free -NH_2_ in non-crosslinked and crosslinked samples, respectively.

### 2.7. Characterization of Structures, Compositions, and Morphologies

Before and after modification, the crystal structure of palygorskite was studied and characterized by X-ray diffraction (XRD, Rigaku, D/max-2550VB+/PC, Tokyo, Japan). The structural details of SNF were characterized by AFM (Bruker Dimension ICON, Karlsruhe, BW, Germany). The chemical composition of PGS, the pure SNF membrane, and the composite membranes were investigated by a Nicolet 8700 FT-IR spectrometer (attenuated total reflectance mode, Thermo Fisher Scientific, Waltham, MA, USA) and X-ray photoelectron spectroscopy (XPS, Escalab 250Xi, Thermo Fisher Scientific, Waltham, MA, USA). The p-PGS and PGS and the surface and cross-sectional morphology of the composite membrane were revealed by field emission scanning electron microscopy (FE-SEM, Hitachi, SU8010, Tokyo, Japan). Because SNF and PGS are nonconductive to electrons, and to improve the image quality, the samples were coated with a conductive layer of sputtered platinum. In the SEM photos, the thickness of the composite membranes was analyzed using the ImageJ2 image visualization software developed by the National Institute of Health (“http://rsb.info.nih.gov/ (1 December 2022)”). The zeta potential of composite membrane surfaces (solution pH of 6.5) was characterized via a solid surface flow potential test system (Anton Paar, SurPASS 3, Graz Austria).

### 2.8. Separation Performance Evaluation of Composite Membranes

The permeation performance of composite membranes was evaluated by measuring the pure water flux (J) across the membrane at 25 ℃ on an ultrafiltration apparatus.

The permeation flux was calculated via Equation (2) as defined below:

(2) $$J=\frac{V}{Atp}$$

where *V* (L) is the volume of permeated water, *A* (m^2^) is the effective membrane area for water permeation, *t* (h) is the permeation time, and *p* (bar) is the applied pressure across the membrane.

Rejection (*R*) for 100 mg L^−1^ dye solution was investigated under an applied pressure of 1 bar. The concentrations of dyes were measured using a UV-Vis spectrophotometer (PERSEE, TU-1950, Beijing, China). The rejection (*R*, %) was calculated using Equation (3):

(3) $$R\;\left(\%\right)=1-\frac{C_{p}}{C_{f}}\times 100$$

where *C_f_* (g L^−1^) and *C_p_* (g L^−1^) represent the concentrations of feed solution and permeate solution, respectively.

## 3. Results and Discussion

### 3.1. Characterization of PGS and SNF

**Figure 2:**
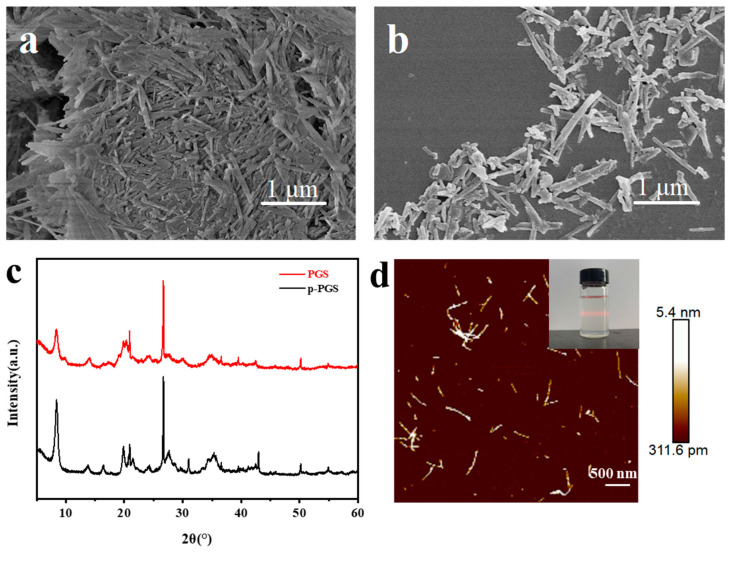
Structural and morphological characterization of p-PGS, PGS, and SNF: (**a**,**b**) SEM images of p-PGS and PGS; (**c**) XRD patterns of p-PGS and PGS; (**d**) AFM image of SNF. The inset is the SNF solution with an obvious Tyndall effect.

Here, the PGS powder was pretreated by acid washing and sintering to remove soluble and organic purities. Figure 2a,b present the SEM images of the p-PGS and PGS. From the SEM images, p-PGS and PGS showed a typical rod-like morphology with diameters of 40–70 nm and lengths of 450–800 nm. The size distribution of p-PGS is relatively uniform, but it is prone to agglomeration. The surface of PGS is rough, and its dispersion is improved. This shows that the surface area can be increased, and the agglomeration can be improved.

The p-PGS and PGS were characterized by XRD, and the characterization results are shown in Figure 2c. The p-PGS is a very crystalline clay. The matrix space of the p-PGS skeleton has an interlayer distance (d = 10.4 Å), so there was a peak at 2θ = 8.5° [33]. The character peaks of 14.0°, 16.4°, 19.9°, and 20.9° represent the Si-O-Si crystalline layers in the clay [34]. After acidification and sintering, the crystallinity of p-PGS was lost. This was most likely due to the decrease in the interlayer spacing of the structure resulting from the leaching of octahedral magnesium [33]. In addition, there are two other peaks at 19.9° and 20.9° in the XRD pattern of PGS; they may be related to the crystal arrangement of Si-O-Si. Like quartz impurities (2θ = 26.7°), they are not eliminated by this method [33]. According to the XRD pattern, the modified treatment does not change the position of the characteristic diffraction peak. Only the intensity of the characteristic diffraction peak changes slightly, that is, the modified treatment does not change the internal crystal structure of attapulgite. 

After incubation at 60 °C for one week, SNFs were obtained by the self-assembly of silk fibroin solution. The obtained SNF solution is homogeneous, transparent, and stable, with an obvious Tyndall effect (inset Figure 2d), and it can be stably stored for 1 month at 4 °C. As can be seen from the AFM image (Figure 2d), the diameter of the SNFs is about 5 nm, and the length is generally greater than 500 nm, which is larger than the pore size of PA filter membranes (450 nm), to ensure that SNFs can be well deposited on PA filter membranes to form the filter cake layer.

This section may be divided by subheadings. It should provide a concise and precise description of the experimental results, their interpretation, as well as the experimental conclusions that can be drawn.

### 3.2. Optimization of Crosslinking Conditions for SNF-PGS-G Membranes

At present, there are mainly three regular aggregation forms of regenerated silk protein that have been reported, which are Silk I, II, and Ⅲ. It is well known that silk protein in dilute solution mainly exists in the form of a random thread cluster (Silk I), but the β-sheet (Silk II) is the most stable configuration of the silk protein’s molecular chain in thermodynamics [35,36]. Therefore, under certain external conditions (such as temperature, pH, crosslinking reagents, etc.), filament proteins can be easily induced to fold from a helical/random thread to a β-sheet [37]. The structure of Silk I is mostly stabilized by intramolecular hydrogen bonds, so Silk I’s structure is water soluble. Silk II, on the other hand, is insoluble in water as the “physical crosslinking point” formed by the β-sheet, which is mainly dependent on the hydrogen bonds between molecules. The transition from Silk I to Silk II is much easier, because it forms a more stable β-sheet.

**Figure 3:**
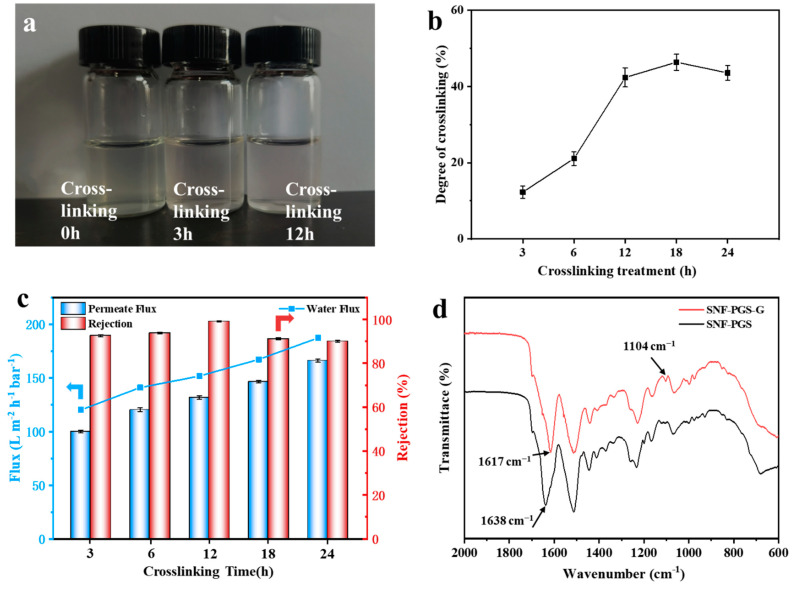
Characterization of SNF-PGS solution and membranes before and after crosslinking: (**a**) visual appearance of SNF-PGS solution for different crosslinking times; (**b**) degree of crosslinking obtained for SNF-PGS solution for the different reaction conditions; (**c**) separation performance of the different crosslinking time of the SNF-PGS (15:1) composite membranes for water and 100 ppm CR solution; (**d**) FT-IR spectrum of SNF-PGS and crosslinked with genipin.

The mixed-dimensional SNF-PGS nanocomposites were prepared by directly mixing different weight ratios of PGS and SNF solution. The formation of covalent bonds in mixed systems can produce stable and ordered materials, which has beneficial effects on their properties. At present, crosslinking agents commonly used include synthetic chemicals such as glutaraldehyde [28,38], epoxy compounds [39], etc., but the silk fibroin materials made of these crosslinking agents have some other disadvantages, such as a certain cytotoxicity [40]. Genipin, as a new crosslinking agent, has been widely used in a variety of macromolecules such as gelatin, collagen, and chitosan. Its crosslinking effect is comparable to that of the traditional chemical crosslinking agent glutaraldehyde, but its cytotoxicity is much lower than that of glutaraldehyde crosslinking materials [27,41]. Therefore, genipin was used to crosslink the SNF structures that make up the majority of the composite membranes. After 3 h of crosslinking, the color of the solution changed from transparent to light blue, indicating the occurrence of the crosslinking reaction (Figure 3a). With the extension of the reaction time, the color of the mixed solution gradually became darker. Genipin can spontaneously react with amino acids or proteins to form a dark blue pigment that is associated with the polymerization of genipin’s free radicals [41,42]. The degree of crosslinking was calculated (Figure 3b).

It can be seen from Figure 3b that when the crosslinking time exceeds 12 h, the change in crosslinking degree is no longer obvious. However, A. Vasconcelos et al. [43] showed that it took 6 h to complete the maximum crosslinking when genipin was used as a crosslinker. On the one hand, in this study, the inorganic substances dispersed in the mixture did not participate in the crosslinking reaction, and the crosslinking time was prolonged. On the other hand, the low solubility of SNF used in this system will also affect the rate of crosslinking reaction. The use of genipin is more time-consuming than other crosslinkers, which may be related to the fact that genipin is mainly associated with lysine and arginine (in protein-based structures). The proportion of lysine and arginine in silk protein was low, about 0.6%, mainly in the hydrophilic blocks. For this reason, the number of crosslinking sites is small, which results in lower crosslinking degrees, and the reaction kinetics should be reasonably slow, but efficient enough to render the system stable in water [44].

Based on the above, crosslinking time has a great impact on the filtration performance of the membranes, because, under different crosslinking times, the main action mechanism of the composite membrane may change. For better control variables, composite membranes with any fixed component ratio, such as SNF-PGS (15:1), were selected in this study to compare their filtration performance at different crosslinking times. Figure 3 directly illustrates the effect of genipin’s crosslinking reaction time and the filtration performance of SNF-PGS composite membranes. As can be seen from Figure 3c, the water flux of the composite membrane increased with the extension of the crosslinking time. The water flux was only 117.5 L m^−2^ h^−1^ bar^−1^ at 3 h of crosslinking and 183.16 L m^−2^ h^−1^ bar^−1^ after 24 h of crosslinking. Congo red (CR), a typical anionic dye, was used to characterize the separation properties of the composite membranes. The variation trend of the permeate flux of CR dye was consistent with that of water, indicating that the performance of the composite membranes was stable in different water environments. As the crosslinking time increased from 3 h to 24 h, the permeate fluxes of the composite membrane to 100 ppm CR solution were 100.15 L m^−2^ h^−1^ bar^−1^, 120.25 L m^−2^ h^−1^ bar^−1^, 131.78 L m^−2^ h^−1^ bar^−1^, 146.49 L m^−2^ h^−1^ bar^−1^, and 166.16 L m^−2^ h^−1^ bar^−1^, respectively. The rejection rate of CR could reach more than 90%, and the highest rejection rate was 98.74% when the crosslinking time was 12 h. In general, as the degree of crosslinking increases, the composite membrane will become more compact, and its permeability should decrease, which is not consistent with the results here. The reaction between -OH in genipin and -NH_2_ in SNF was incomplete, and strong intermolecular hydrogen bonds were formed easily between SNF-SNF and SNF-genipin, resulting in poor water permeability of the composite membrane [45,46] when the crosslinking reaction time was insufficient. The increase in crosslinking degree not only weakens the intermolecular hydrogen bond, but also makes the composite membrane more stable. Therefore, the permeability of the composite membrane was improved. When the crosslinking time exceeds 12 h, the degree of crosslinking had no effect, and the composite membranes were mainly affected by the trade-off effect. When no crosslinking agent was added, the rejection of the SNF-PGS composite membrane for 100 ppm CR solution was 88.3%. As such, compared to not being crosslinked, the selectivity of CR separation was also improved, and when the crosslinking time is 12 h, the selectivity of the composite membrane is the best. Therefore, composite membranes with a crosslinking time of 12 h were selected for further characterization, and in the following, they are denoted as SNF-PGS-G membranes.

The FT-IR spectrum of SNF ranged from 600 to 2000 cm^−1^, as shown in Figure 3d. For silkworm silk fibroin, its infrared spectrum showed that the absorption peaks of Silk I appeared near 1638 cm^−1^ (amide I), 1513 cm^−1^ (amide II), 1233 cm^−1^ (amide III), and 680 cm^−1^ (amide V). The absorption peaks of Silk Ⅱ appeared near 1620 cm^−1^ (amide I), 1521 cm^−1^ (amide II), 1230 cm^−1^ (amide III), and 695 cm^−1^ (amide V). The absorption peaks of the random coil structure appeared at 1650–1660 cm^−1^, 1535–1545 cm^−1^, 1235 cm^−1^, and 650 cm^−1^. As can be seen from Figure 3d, the uncrosslinked SNF spectra show the bands of amide I at 1638 cm^−1^ (C=O stretch), amide II at 1513 cm^−1^ (N-H deformation), and amide III at 1233 cm^−1^ (C-N stretch, C=O bending vibration), indicating the random coil/Silk I conformation [43,47,48]. Genipin crosslinking and methanol treatment rearranged SNF structure and induced SNF to form β-sheet conformation. By comparison, the characteristic peak of amide I (1617 cm^−1^) shifted to a lower wave number with the increase in genipin, confirming the transition from random coil to β-sheet in the SNF membranes [47,48,49]. In addition, a new absorption peak appeared at 1104 cm^−1^(-COH), which may be due to the formation of a new covalent bond after genipin was crosslinked with SNF [43].

### 3.3. Optimization of Mass Ratio for SNF-PGS-G Membranes

**Figure 4:**
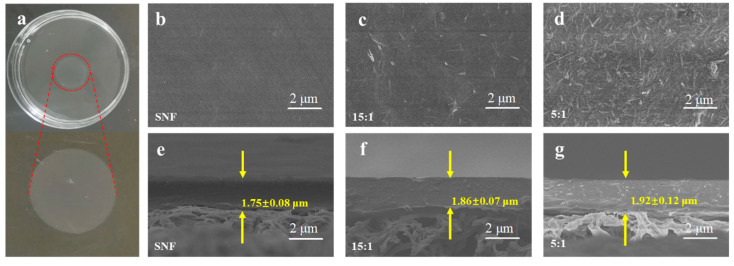
Visual appearance and structural characterization of the obtained membranes: (**a**) photos of SNF-PGS-G composite membranes with a transparent and uniform surface; (**b**–**g**) surface and cross-sectional scanning electron microscope (SEM) images of the SNF-G, SNF-PGS-G (15:1), and SNF-PGS-G (5:1) membranes.

After determining the uniform crosslinking time, the composite membranes were prepared by vacuum filtration of SNF-PGS-G mixtures with different mass ratios. As the control, pure SNF membranes were prepared by the same procedure. The assembly of the composite membranes is simple. Figure 4a shows that the macroscopic composite membranes, which can be peeled off from the substrates, are self-supporting, stable, and have a transparent and uniform surface. SEM was used to characterize the morphology and microstructure of the membranes. Figure 4 shows the observed results of SNF-G, SNF-PGS-G(15:1), and SNF-PGS-G(5:1) membrane surfaces and cross sections. The results showed that the surface of the pure SNF membrane was relatively uniform and dense, and the surface of composite membranes became uneven with the increase in PGS composition. As can be seen in Figure 4c,e the rod-like structure of PGS was uniformly and irregularly dispersed in the composite membranes, of which only a small part floats on the surface, and most of them are wrapped by SNF. It could be concluded that the cross-section of the pure SNF membrane was tighter and more compact. However, the SNF-PGS-G composite membranes became looser. The thickness of the SNF-G, SNF-PGS-G(15:1), and SNF-PGS-G(5:1) membranes was 1.75 ± 0.08 μm, 1.86 ± 0.07 μm, and 1.92 ± 0.12 μm, respectively. With the increase in the mass ratio of PGS, the thickness of the composite membranes increased somewhat, probably because the size of SNF (~5 nm) is much smaller than the size of PGS. The thickness of composite membranes with different mass ratios was 1.65–2.05 μm, because the same theoretical deposition amount was used in the process of membranes made under different SNF-PGS-G mass ratios. What is more, due to the treatment of p-PGS before membrane preparation, PGS is dispersed in the membrane, which overcomes the agglomeration phenomenon in the early stage.

**Figure 5:**
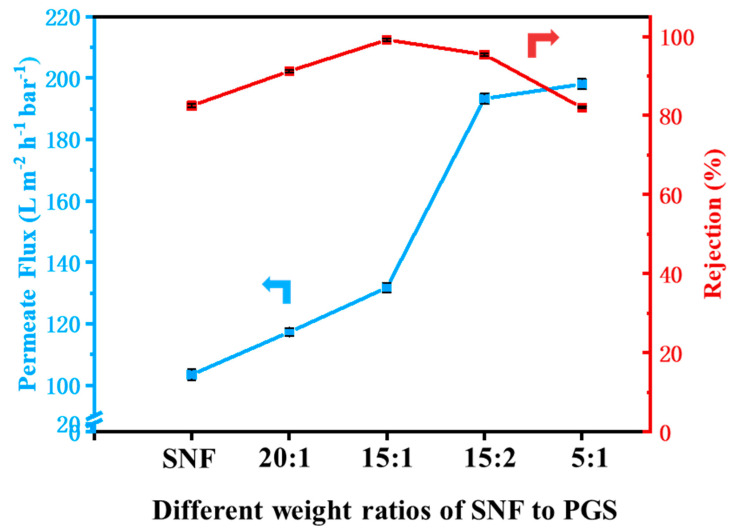
Separation performance of the different weight ratios of the SNF-PGS-G composite membranes for 100 ppm CR solution.

The effect of SNF/PGS mass ratios on the filtration performance of SNF-PGS-G composite membranes is shown in Figure 5. For CR, the rejection rate can reach 99.16%. A remarkable phenomenon is that the flux of the composite membrane increases with the addition of PGS content. The intercalation of PGS nanorods into SNF nanofibers can simultaneously tailor the channel structure and surface topography of the as-prepared SNF-PGS-G membranes, endowing membranes with hierarchical nanostructures and enhanced water transport performance. The addition of the hydrophilic substance PGS effectively enhanced the hydrophilicity of the SNF-PGS-G composite membranes [25]. At the same time, due to the large specific surface area, good ionic exchangeability, and abundant surface groups [50,51], PGS could adsorb dyes inside the pores, so an adsorption-assisted filtration process was proposed for the SNF-PGS-G membranes to separate dyes. It should be emphasized that SNF-PGS-G composite membranes do not need to be used as adsorbents to remove dye for a long time like traditional adsorption, but can be directly used as a filter for rapid separation of the dye. However, too much PGS not only meant it was not easy to disperse the membranes evenly, but also indicated that the pores in the composite membranes would be too large and could not achieve effective separation of the dye. It can be seen from Figure 5 that the composite membranes with a mass ratio of SNF/PGS of 15:1 had better filtration performance for CR cationic dyes. Therefore, the SNF-PGS-G(15:1) composite membranes were determined for subsequent characterization and experiments.

**Figure 6:**
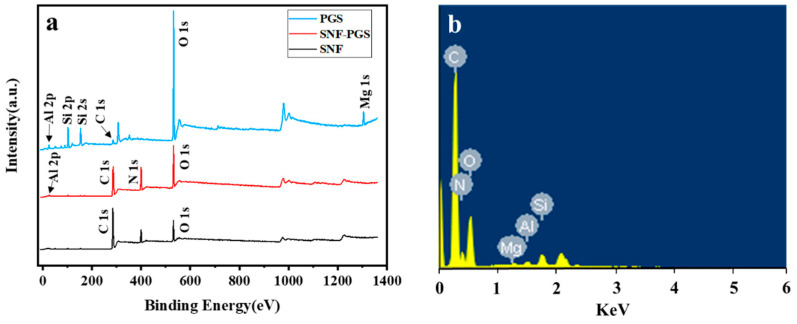
Elemental compositions of membranes: (**a**) XPS patterns of PGS, SNF, and SNF-PGS (15:1) composite membranes; (**b**) SEM-EDS spectrum of SNF-PGS (15:1) composite membranes.

XPS was used to reveal the chemical species of elements in the surface region of PGS, SNF, and SNF-PGS (15:1) composite membranes (Figure 6). All three materials showed C 1s (285 eV), O 1s (525 eV), Si 2s (153 eV), and Si 2p (102 eV). Moreover, the PGS aluminum and magnesium species can be characterized by Al 2p and Mg 1s located at approximately 96 eV and 1304 eV. The N element in SNF is reflected by N 1s (400 eV) and is also reflected in the XPS spectrum of SNF-PGS (15:1), indicating the existence of SNF in the composite membranes. The O/N ratios and elemental compositions of the three materials are different. The O/N ratio was 1.017 in pure SNF membranes, and it increased to 1.142 in composite membranes. Among them, all N comes from SNF. The increase in the O ratio in the composite membrane suggested that there might be additional O sources, namely, PGS, other than that from the SNF composition. As the content of PGS in the SNF-PGS (15:1) composite membrane is small, as shown in Table 1, Mg and Al account for only 3.13% and 5.51% of PGS powder, respectively. Further, the detection thickness of XPS is about 5 nm on the membrane surface. However, in the prepared membrane, the content of PGS is relatively low (~6.6%), and the content of Mg in PGS is 3.13%. Due to the large thickness of the membrane prepared by vacuum-assisted filtration, as shown in Figure 4c, the rod-like structures (PGS) on the membrane surface are dispersed, which made it difficult to detect Mg and Al in the XPS spectrum of the composite membranes. In this regard, the presence of PGS was demonstrated by the signals of Mg and Al elements in the elemental mapping and the SEM-EDS spectrum of the composite membranes.

### 3.4. Filtration Performance of SNF-PGS-G Membranes

**Figure 7:**
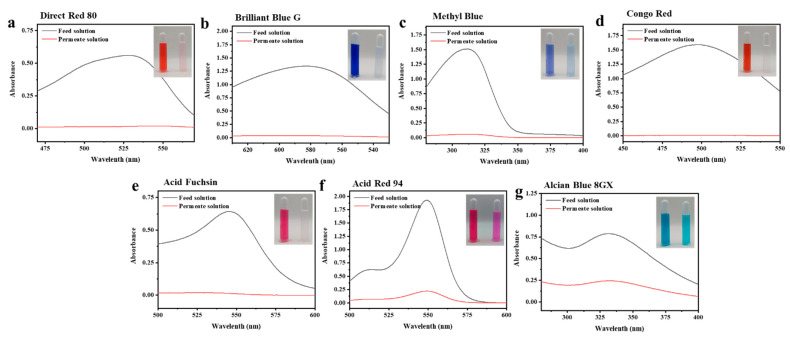
UV−vis absorption changes of an aqueous solution of (**a**) Direct Red 80; (**b**) Brilliant Blue G; (**c**) methyl blue; (**d**) Congo red; (**e**) acid fuchsin; (**f**) acid red; (**g**) Alcian blue 8Gx;.The inserts are comparative photographic images of feed solution (left) and permeate solution (right) of different dyes.

**Figure 8:**
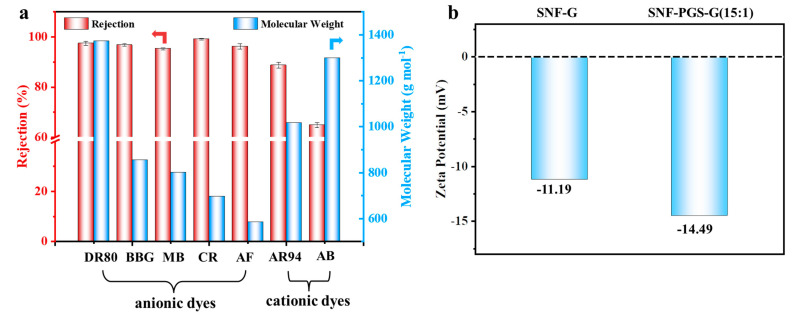
(**a**) The filtration performance of SNF-PGS-G composite membrane for different dyes with different molecular weights; (**b**) zeta potential values of the SNF-G membrane and SNF-PGS-G (15:1) composite membrane.

To further study the filtration performance of the modified SNF-PGS-G composite membrane for various anionic dyes, different dye solutions were selected to test the separation performance of the SNF-PGS-G membranes. For ease of testing, Direct Red 80 (DR80), Brilliant Blue G (BBG), methyl blue (MB), and acid fuchsin (AF) with the same concentration of 100 mg L^−1^ dye solution and 40 mL of the feed solution were filtered through a vacuum filter device, which proved its universality in the separation of anionic dyes. The concentrations of feed solution and permeate solution were monitored by UV-vis spectra. As shown in Figure 7, the concentrations of anionic dyes decreased obviously to a minimum after filtration, and the rejection rate against these anionic dyes and their structures are shown in Figure 8a and Table 2. The rejection rates of different anionic dyes by SNF-PGS-G membranes were a little different. Although the molecular weights of the selected dyes had enormous differences, their rejection rates all remain above 95%. Additionally, the observed rejection of different dyes increased with an increase in the molecular weight of dyes, which is mainly due to the aperture sieving of the SNF-PGS-G composite membranes. Remarkably, even compared with DR80 (Mw = 1373.07 g mol^−1^), the composite membrane showed a greater rejection rate of CR (Mw = 696.66 g mol^−1^), up to 99.16%. This is mainly because CR dye molecules tend to aggregate in water, and their larger molecular size leads to a higher rejection rate [52]. Furthermore, the rejection rate of AF (Mw = 585.54 g mol^−1^) was marginally higher than that of MB (Mw = 799 g mol^−1^), and there is a large probability that the result is within the allowable experimental error. Compared to anionic dyes, two cationic dyes, acid red (AR, Mw = 1017.64 g mol^−1^) and Alcian blue 8GX (AB, Mw = 1298.86 g mol^−1^), were also chosen as target molecules to test the filtration performance of the SNF-PGS-G membranes. This composite membrane exhibited a low rejection rate against cationic dyes, only 88.73% for AR and 64.83% for AB (Figure 7 and Figure 8a). Thus, this implied that for the dyes with small molecular weights (<2000 g mol^−1^), the difference in the filtration performance of the composite membranes to different dyes is mainly due to their charge, rather than their molecular weight. The zeta potential of the SNF-G membrane and SNF-PGS-G(15:1) composite membrane in dye solution (pH = 6.5) was measured (Figure 7c). The negatively charged (zeta potential: −15.49 mV) composite membrane repels the negative anionic dye in the solution, resulting in a higher dye rejection rate. In addition, compared with the SNF-G membrane, the electronegativity of the composite membrane after adding PGS is stronger, which also explains the better anion interception performance of the SNF-PGS-G(15:1) composite membrane.

Table 3 summarizes the data on DR80 or CR dye properties in the literature compared with this study. The results showed that SNF-PGS-G membranes had excellent separation performance. It can be seen from Table 3 that the SNF-PGS-G membrane has superior permeability and a relatively high rejection rate. These results indicated that SNF-PGS-G is a promising choice for the separation of anionic dyes by commercial membranes.

## 4. Conclusions

This paper describes a simple and effective method using self-assembled SNFs doped with PGS to prepare composite membranes to efficiently separate anionic dyes. With an excellent aspect ratio (d: ~5 nm; l: >500 nm; l/d: >100), SNFs in composite membranes can separate dyes well by screening. After adding PGS, a membrane separation system with adsorption-assisted filtration was successfully constructed, which effectively improved the permeability of the membrane. Genipin was used as a crosslinking agent to induce the conformational transition of SNF chains from random coils to β-sheets, which made the composite membranes’ structure more stable and the separation performance more excellent. The separation mechanism of SNF-PGS-G membrane for anionic dyes is proposed as adsorption-assisted pore size filtration. The obtained composite membranes can be self-supported and have good separation performance for organic anionic dyes. Under the optimized conditions of a genipin crosslinking time of 12 h and a SNF to PGS ratio of 15:1, good filtration performance of anionic dyes was achieved with high permeate flux. Specifically, the water flux is up to 148.36 L m^−2^ h^−1^ bar^−1^, and the rejection of common anionic dyes is above 95%, among which the rejection rate of CR is as high as 99.16%. These results proved the great potential of SNF as green materials for the removal or separation of dyes in applications such as wastewater treatment.

## Figures and Tables

**Table 1:** Elemental compositions and oxygen-to-nitrogen (O/N) ratios of PGS, SNF, and SNF-PGS (15:1) membranes from XPS analysis.

Membranes	XPS Surface Elemental Analysis						
	C (%)	N (%)	O (%)	Mg (%)	Al (%)	Else Element	O/N Ratio
PGS	8.53	/	59.63	3.13	5.51	26.33	/
SNF	72.91	12.87	13.09	/	/	1.13	1.017
SNF-PGS (15:1)	58.45	18.77	21.44	0.27	0.32	0.75	1.142

**Table 2:** Characteristics and structures of the dyes used in this study.

Generic Name	Mw	Electric Charge (pH = 6.5)	Molecular Structure
Congo Red	696.66	-	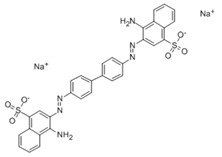
Direct Red 80	1373.07	-	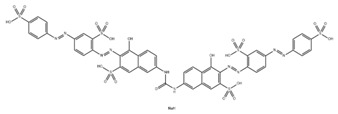
Acid Fuchsin	585.54	-	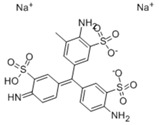
Brilliant Blue G	854.02	-	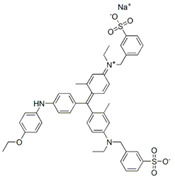
Methyl Blue	799.80	-	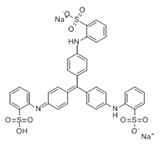
Acid Red 94	1017.64	+	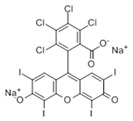
Alcian Blue 8GX	1298.86	+	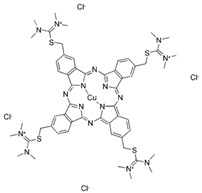

**Table 3:** Performance comparison between the membranes in this work and the reported membranes.

Membranes	Dye	Testing Conditions	Permeate Flux (L m^−2^ h^−1^ bar^−1^)	Rejection (%)	Ref.
GO-TiO_2_/PC	13.7 mg L^−1^ DR 80	1 bar, 25 °C	28.2	76.7	[53]
GO-TiO_2_/P25/PC	13.7 mg L^−1^ DR 80	1 bar, 25 °C	28.2	93.0	[53]
CS–PEO–PTEGDMA/PAN	100 mg L^−1^ DR 80	1 bar, 25 °C	58.8	99.9	[45]
LNFM-2	200 mg L^−1^ CR	4 bar, 25 °C	53.23	99.6	[54]
TiO_2_/PES	100 mg L^−1^ CR	2 bar, 25 °C	65	90	[55]
PVDF/Ag–TiO_2_- APTES	CR	6 bar, 25 °C	18.6	97.4	[56]
TpPa-1/HPAN	500 mg L^−1^ CR	1 bar, 25 °C	41.85	99	[57]
CNTs/LDH	100 mg L^−1^ CR	1 bar, 25 °C	~180	98	[58]
SNF-PGS-G	100 mg L^−1^ DR 80	1 bar, 25 °C	130.3	97.5	This work
SNF-PGS-G	100 mg L^−1^ CR	1 bar, 25 °C	131.8	99.2	This work

## Data Availability

This study did not report any data.
